# Congenital Tracheobronchomegaly (Mounier-Kuhn Syndrome) in a Woman with Human Immunodeficiency Virus: A Case Report

**DOI:** 10.7759/cureus.1136

**Published:** 2017-04-04

**Authors:** Amanda Fletcher, Justin Stowell, Socrates Jamoulis

**Affiliations:** 1 Internal Medicine, Truman Medical Center, University of Missouri School of Medicine, Kansas City, MO, USA; 2 Department of Radiology, Truman Medical Center, University of Missouri School of Medicine, Kansas City, MO, USA

**Keywords:** congenital tracheobronchomegaly, mounier-kuhn syndrome, human immunodeficiency virus, mks, hiv, respiratory infections, hiv-infection, tracheobronchomalacia, recurrent respiratory infections, bronchiectasis

## Abstract

Congenital tracheobronchomegaly (Mounier-Kuhn Syndrome, MKS) is a rare idiopathic disorder characterized by dilation of the central airways, including the trachea and first through fourth order bronchi. MKS disproportionately affects men and results in chronic respiratory tract infections. The diagnosis is made through the synthesis of clinical and radiological data. Here we report a unique case of MKS in a patient with human immunodeficiency virus (HIV) infection. A 45-year-old African American woman with a past medical history of HIV, tobacco and recreational drug abuse, chronic obstructive pulmonary disease, sleep apnea, and a 15-year history of recurrent respiratory infections presented with dyspnea, wheezing, a productive cough, increased yellow-green sputum production, and subjective fevers. Computerized tomography (CT) of the chest revealed striking dilation of the trachea and central bronchi. Fiberoptic bronchoscopy demonstrated a dilated trachea and bronchial tree with complete collapse of the trachea and bilateral mainstem bronchi during expiration. Serial imaging over 14 years allowed the radiologist to confidently diagnose her underlying disorder and recommend appropriate clinical management, which included mucolytics, chest physiotherapy, prophylactic vaccinations, and antibiotics during infectious exacerbations. To the best of our knowledge, there is only one reported case of MKS in the setting of HIV in the English literature. We report the second such case and outline the clinical presentation, diagnostic criteria, and management of MKS with the hope that increased awareness will prevent delayed or misdiagnosis for patients with MKS. This case highlights the common diagnostic delay for MKS and the need to include MKS in the differential diagnosis of recurrent respiratory tract infections.

## Introduction

Congenital tracheobronchomegaly (Mounier-Kuhn syndrome, MKS) is a rare idiopathic disorder characterized by dilation of the central airways, including the trachea and first through fourth order bronchi, and chronic respiratory tract infections. Tracheobronchomalacia and bronchiolectasis, beyond the fourth order bronchi, are associated morbid conditions that constitute a challenge for treatment. Reports of enlarged airways have been described dating back to 1897 [1] with the first clinical description of the disease by Mounier-Kuhn in 1932 [2]. To date, fewer than 400 cases of MKS have been reported [3-6]. While no epidemiologic study has been published, MKS has been found to disproportionally affect men with an 8:1 male to female ratio [4-5]. There is also a prevalence of MKS in both smokers and African-Americans [3], and patients typically present in the third through sixth decades of life [3,5]. The diagnosis is made through the synthesis of clinical and radiological data. Here we report a unique case of MKS in a patient with human immunodeficiency virus (HIV) infection. To the best of our knowledge, this case represents the second documented patient with concomitant MKS and HIV [3]. Informed consent was waived as all information presented is de-identified.

## Case presentation

A 45-year-old female with a past medical and social history of HIV diagnosed at age 32, high-risk sexual activity, tobacco and recreational drug abuse, chronic obstructive pulmonary disease (COPD), sleep apnea, and a 15-year history of recurrent respiratory infections presented with dyspnea, wheezing, a productive cough, increased yellow-green sputum production, and subjective fevers. Tachycardia, bronchial breath sounds, diffuse expiratory wheezing, and rhonchi were noted on physical examination. Laboratory analysis was significant for leukocytosis (white blood cell count of 12.0 × 10^3^/µL) with normal procalcitonin (<0.05 ng/mL). Potassium hydroxide preparation, acid-fast bacteria stain, Streptococcus and Legionella antigens, and Mycoplasma pneumoniae antibody IgM, as well as fungal, bacterial, and viral respiratory cultures were all negative.

Her respiratory symptoms had escalated in 12 months prior to this admission, leading to four hospital admissions for pneumonia and respiratory failure, two of which required intensive care unit admission. She suffered recurrent COPD exacerbations, often treated with outpatient antibiotics. Serial spirometry dating back seven years showed obstructive physiology, but was normal on this admission. She had been an established patient in the infectious disease and pulmonology clinics for 10 years.

**Figure 1:**
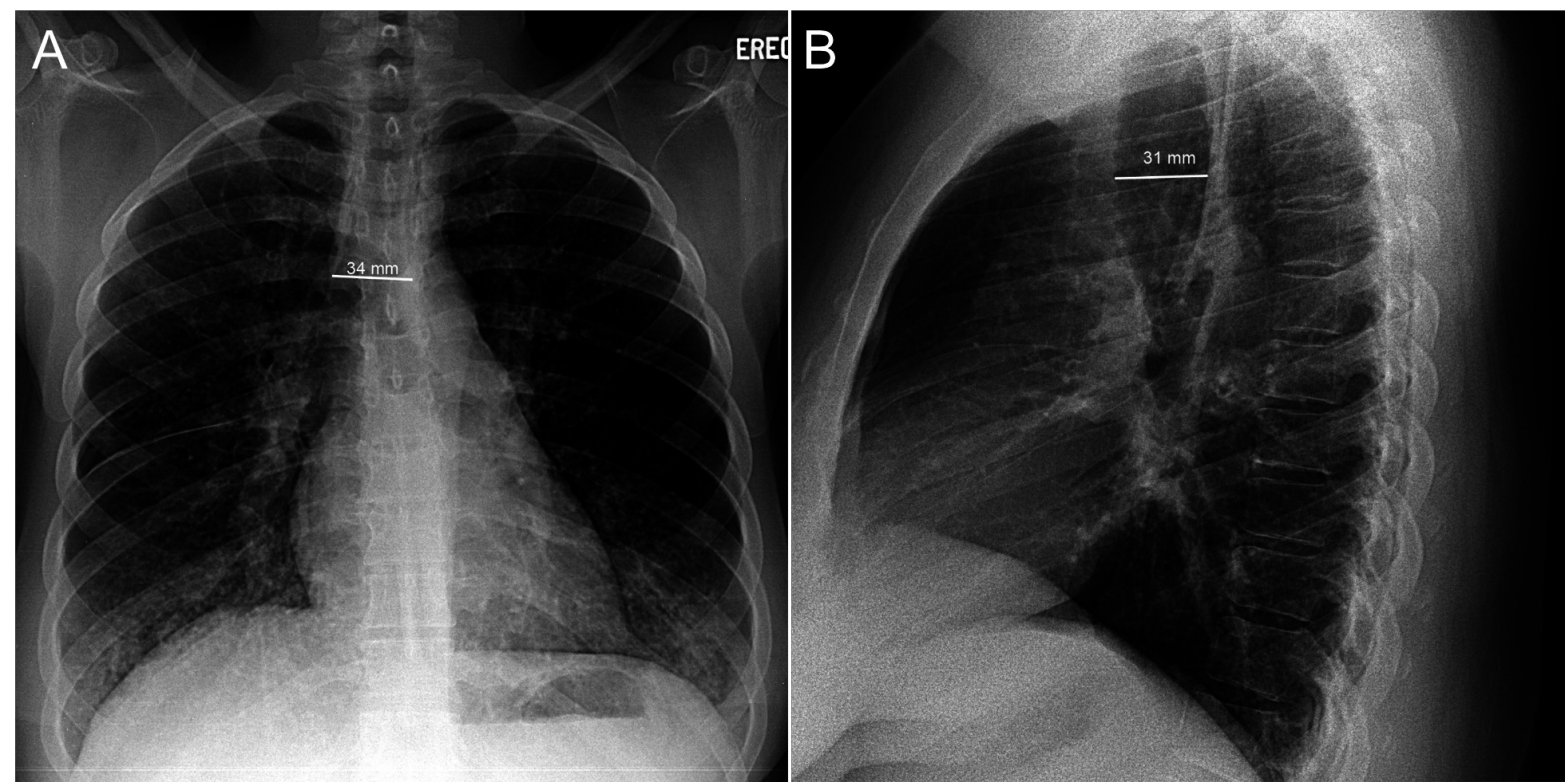
Posterior-anterior (A) and lateral (B) chest radiographs Chest radiographs from fourteen years prior demonstrate unchanged, chronic tracheobronchomegaly.

**Figure 2:**
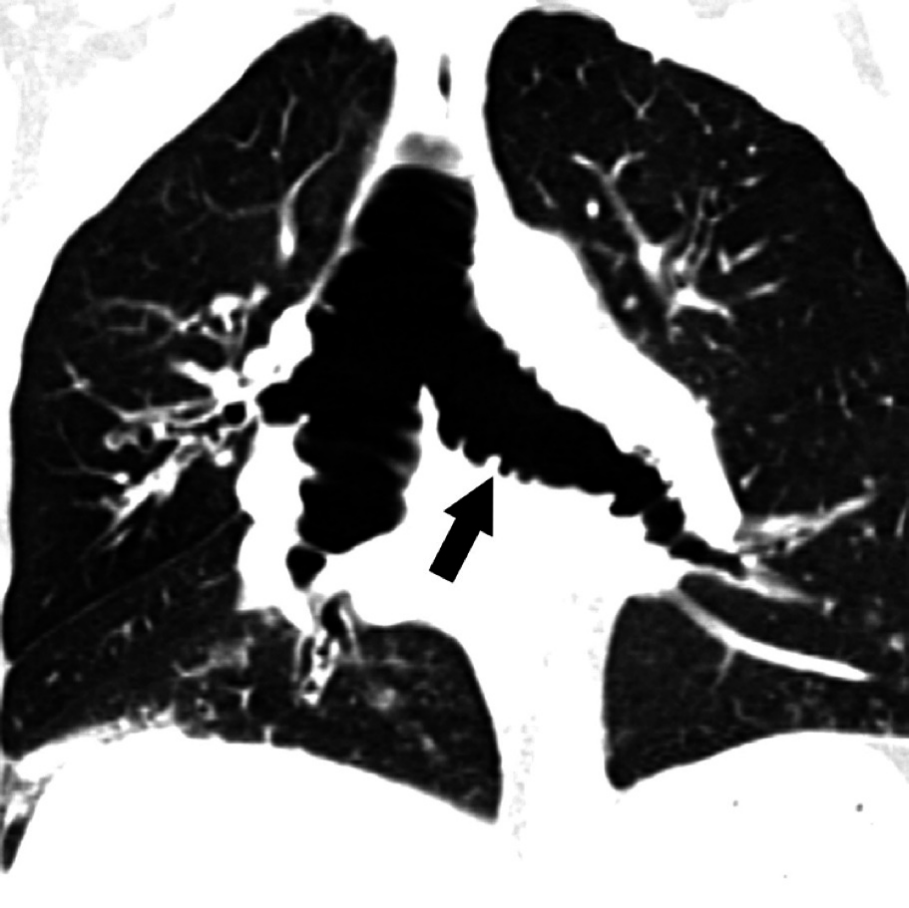
Contrast-enhanced coronal computerized tomography of the chest Computerized tomography demonstrates dilation of the trachea and central bronchi. The central airways exhibit a corrugated appearance related to prolapsing, redundant mucosa (arrow). Scattered tracheal diverticula are also seen.

**Figure 3:**
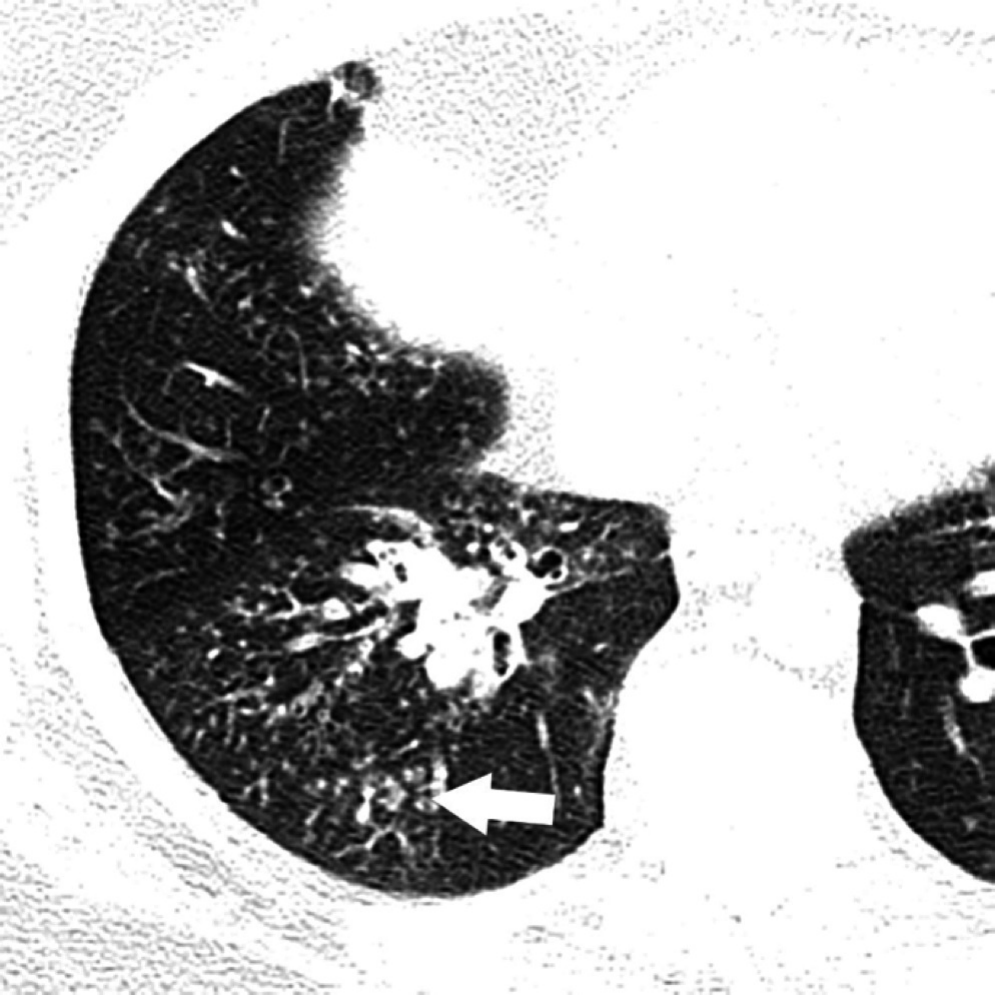
Axial computerized tomography of the chest Computerized tomography of the chest reveals right middle lobe and right lower lobe tree-in-bud opacities (arrow), consistent with infectious bronchiolitis.

**Figure 4:**
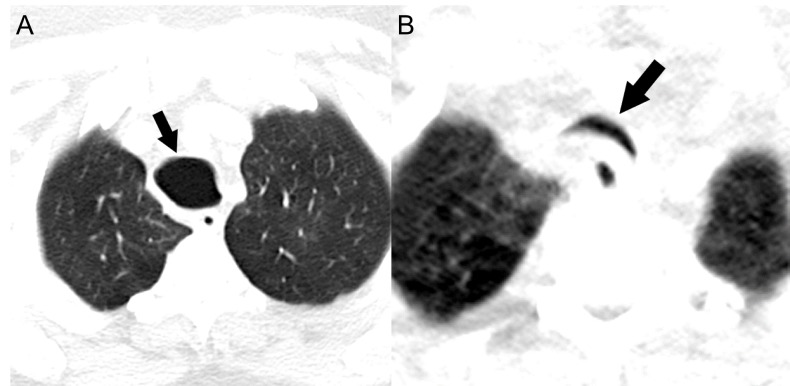
Inspiratory (A) and expiratory (B) computerized tomography of the chest Computerized tomography of the chest confirms tracheomegaly and associated tracheobronchomalacia. Tracheobronchomalacia is evidenced by the lunate shape of the trachea on inspiration (arrow, A) and near-complete collapse of the trachea with a 'frown sign' (arrow, B) on expiration.

On chest radiographs, the posterior-anterior diameter of the trachea measured 34 mm and the lateral diameter 31 mm, unchanged over 14 years (Figures 1A-1B). The posterior-anterior diameter of the right and left main bronchi measured 24 mm and 16 mm, respectively. Computerized tomography (CT) of the chest revealed striking dilation of the trachea and central bronchi, with scattered tracheal diverticula and sacculations giving a corrugated appearance (Figure 2). Diffuse segmental cylindrical bronchiectasis and bronchiolectasis were present. Multifocal consolidation and tree-in-bud opacities were also seen, consistent with infectious bronchiolitis (Figure 3). Marked (>70%) tracheal luminal diameter collapse was visualized retrospectively on expiratory phase imaging from a prior CT neck examination, indicating associated tracheobronchomalacia (Figure 4). These findings had been present though inconsistently reported on prior examinations, and never clinically addressed.

Fiberoptic bronchoscopy demonstrated a dilated trachea and bronchial tree. There was a complete collapse of the trachea and bilateral mainstem bronchi during expiration, diagnostic of tracheobronchomalacia. Mucous plugs and circumferential tracheal and main bronchial diverticulae were noted centrally, not involving the subsegmental bronchi. Together, these findings along with serial imaging over 14 years allowed the radiologist to confidently diagnose her underlying disorder as MKS. While hospitalized, her symptoms improved after antibiotics, oral steroids, respiratory support, and chest physiotherapy. As an outpatient, she is currently well managed with mucolytics, chest physiotherapy, prophylactic vaccinations, and antibiotics during infectious exacerbations.

## Discussion

MKS is a rare congenital disorder of the central airways characterized by abnormal tracheobronchial dilation. This is distinguished from acquired tracheal dilation described in rheumatoid arthritis, pulmonary fibrosis, ankylosing spondylitis, and other conditions. The diagnosis of MKS is made through the synthesis of both clinical and radiological data. Non-specific clinical features typically include recurrent respiratory tract infections; chronic, loud, productive cough; dyspnea; and hemoptysis. Pulmonary function tests commonly show increased dead space, total lung capacity, residual volume, and obstructive physiology related to large airway collapse. However, pulmonary function tests have also been reported as normal [7] as seen in our patient. A broad spectrum of clinical courses has been documented in MKS, ranging from minimal disease with good preservation of pulmonary function to progressive disease leading to respiratory failure and death [8]. Our patient experienced clinical progression of her respiratory symptoms; however, her tracheobronchomegaly remained unchanged in size for 14 years on imaging.

While tracheobronchomegaly can be detected on chest radiography, it is commonly under-recognized as evidenced by this case. In adults, tracheobronchomegaly is diagnosed on chest radiography and CT when the coronal tracheal diameter (measured 2 cm above the carina) exceeds 30 mm, or wider than the superimposed thoracic vertebral bodies. In addition, coronal diameters of the right and left main bronchi should measure greater than 21 mm and 18 mm for men and 20 mm and 17 mm for women, respectively [8]. Diverticulae along the length of the trachea and central bronchi produces a corrugated appearance on imaging (Figure 2). Dynamic inspiratory and expiratory CT helps confirm tracheobronchomalacia commonly present in these patients. Radiographic findings, including dynamic airway collapse and diverticula, may be confirmed with bronchoscopy. Pathologic hallmarks of MKS include thinning of the muscularis mucosa, atrophy of longitudinal muscle and elastic fibers, and absence of the myenteric plexus of the involved airways [9]. Our patient had radiographic evidence of MKS for 14 years before it was consistently reported and clinically addressed.

Conservative management using mucolytic agents and chest physiotherapy, including massage and postural drainage, are the mainstays of treatment [3,10]. The pneumococcal polysaccharide and influenza vaccines are recommended regardless of age and symptomatology [3]. There are no definitive prospective data supporting prophylactic antibiotic use. However, acute exacerbations should be managed using guidelines for non-cystic fibrosis bronchiectatic disease and lower respiratory tract infections. Infection with atypical organisms, including tuberculous and non-tuberculous mycobacteria, may complicate some cases [5]. Control and prevention of recurrent infections will prevent progression to irreversible pulmonary fibrosis. Several trials have also shown benefit of continuous positive airway pressure, airway stenting, and tracheobronchoplasty [9].

## Conclusions

To the best of our knowledge, this represents the second reported case of MKS in the setting of HIV. Additionally, this female patient is exceptional in that MKS is almost exclusively found in males. While our patient experienced clinical progression of her respiratory symptoms, her tracheobronchomegaly remained unchanged in size for 14 years on imaging. Serial imaging over 14 years allowed the radiologist to confidently diagnose her underlying disorder and recommend appropriate clinical management, which included mucolytics, chest physiotherapy, prophylactic vaccinations, and antibiotics during infectious exacerbations. Although her HIV has been well-managed, clinical management of future exacerbations could lead to complicated and extensive infectious disease workups. This case highlights the common diagnostic delay for MKS and the need to include MKS in the differential diagnosis of recurrent respiratory tract infections.
